# Social justice and social media: How medical schools display critical consciousness online

**DOI:** 10.1371/journal.pdig.0000981

**Published:** 2025-08-07

**Authors:** Eray Yilmaz, Keegan D’Mello, Amrit Kirpalani

**Affiliations:** 1 Department of Paediatrics, Schulich School of Medicine and Dentistry, Western University, London, Ontario, Canada; 2 London Health Sciences Centre Research Institute, London, Ontario, Canada; Emory University, UNITED STATES OF AMERICA

## Abstract

Academic medical institutions have a pivotal role in addressing the inequalities faced by marginalized populations, especially by promoting values of social justice on online platforms that not only reach the medical sphere, but also the broader public. Central to this transformative agenda is the framework of critical consciousness (CC), which compels individuals to develop an acute awareness of societal inequalities and power dynamics and act as agents of change against inequalities across society. To investigate if and how medical schools use X (formerly Twitter) to display CC, tweets from March 22 – June 22, 2023 from all available Canadian medical school Twitter accounts were obtained and deductively coded. First, a content analysis was performed to collate and categorize tweets related to CC, followed by a critical discourse analysis with a CC framework to examine the role of language in conveying messages about equity and medical education. Of the 3442 tweets reviewed, 554 displayed CC (16.12%). The content analysis revealed that Empowerment of Marginalized Populations was the most prominent display of CC amongst tweets (n = 286), whereas there was a paucity of messaging around Intersectionality (n = 20). The critical discourse analysis revealed that language was purposefully used to positively spotlight equity-deserving individuals (e.g., “celebrate” and “recognize”) with minimal dialogue framing institutions as agents of systemic power differentials. Medical schools ultimately advocate for positive change by sharing awareness-raising content that celebrate marginalized communities. However, the step beyond surface-level awareness-raising content towards critical self-reflection that acknowledged institutions’ roles in perpetuating inequities was largely limited; this represents a missed opportunity to leverage the power of social media and engage in meaningful dialogue online to build trust between the healthcare sector and the public.

## Author summary

In our study, we explored how Canadian medical schools use the social media platform X (formerly Twitter) to share messages of social justice and equity. Social media offers these institutions a powerful tool to raise awareness of systemic inequities and foster dialogue. By analyzing over 3,400 tweets from official accounts, we found that while many posts highlighted the achievements of marginalized groups and celebrated diversity, fewer delved into critical self-reflection about the schools’ own roles in perpetuating inequities. This emphasis on celebration over introspection reveals a missed opportunity. Social media can serve as more than just a platform for awareness; it can foster meaningful dialogue about systemic change. By acknowledging their positions of power and addressing internal challenges transparently, medical schools can build trust with the public and the communities they aim to serve. Our findings underscore the importance of moving beyond surface-level advocacy. Embracing deeper, reflective engagement online could strengthen the social accountability of medical institutions, ultimately advancing the broader goal of equitable healthcare.

## Introduction

With recognition of escalating health disparities, academic medical institutions have a pivotal role in advocating for systemic change, particularly in addressing inequalities faced by marginalized populations. This mission is critical for medical schools, which not only educate the next generation of healthcare professionals but also hold significant influence in public discourse on health justice and promote equity, diversity, and inclusion in their mission [1]. The language employed by medical institutions has the ability to reinforce existing norms or drive change, and therefore have a fundamental role in shaping health systems and practices. Public discourse by medical schools gives insight into how institutions “conceptualize social problems” in medicine and their commitment to health equity [2]. These schools have a unique opportunity to use their platform to reflect their values and promote social justice. Acknowledging their privilege and uplifting marginalized communities will help spearhead transformative change in the medical community [3].

Central to this transformative agenda is the concept of Critical Consciousness (CC), a framework for understanding and acting against systemic injustices. Introduced by Paulo Freire in “Pedagogy of the Oppressed,” CC compels individuals to recognize the roots of social, political, and economic inequalities and become active agents of change [4]. Watts et al. operationalize CC into three foundational components:

1. Critical reflection – the ability to analyze inequities in society’s culture, practices, and policies
2. Political efficacy – the perceived ability to instigate sociopolitical change
3. Critical action – engaging in behaviours to dismantle and challenge oppressive societal structures.

While some scholars also incorporate *critical motivation* as a distinct driver of equity-oriented efforts, highlighting the importance of emotional and personal commitment in participants of social change, this study focuses on the foundational three-component model [5,6]. In the realm of healthcare, CC fosters an understanding (i.e., critical reflection) among professionals of how systemic issues, like poverty, impact health outcomes [7]. Greater awareness of root cause of health disparities can empower healthcare professionals to challenge institutional norms (i.e., political efficacy), urging healthcare workers to use their influence to advocate for policies that dismantle oppression and promote equity (i.e., critical action) [5].

The increasing use of social media by medical schools represents a novel avenue for advocating CC principles. Social media represent a significant space for diverse discourse, offer medical institutions the means to share their values and initiatives, impacting not only their immediate academic community but also the broader public sphere [8–11]. Although messages are limited to 280 characters, X (formerly known as Twitter) proves to be unique social media platform as it allows for the rapid spread of concise information which has the power to drive societal changes in real time. Unlike other social media platforms like Instagram which primarily use visual messaging as a form of communication, tweets involve linguistic, text-based content that can be aggregated under trending topics and hashtags to illustrate user activity related to specific subjects. Papacharissi (2014) discusses how storytelling and shared sentiments expressed on X draws public attention to current sociopolitical issues or movements. For example, 600 000 tweets analyzed from the #MeToo movement showcases how collective social media messaging empowered marginalized voices to share personal experiences and raise awareness about sexual violence [12–15]. Sociological analyses by Murthy (2024) further emphasizes that X serves as a critical site for public discourse and the sociological insights about the social world on X are a transferable framework to study any future platform. Hence, findings derived from X research provide a structure to understand future platforms, underscoring the relevance of examining institutional messaging on X to assess critical consciousness [15]. Despite the potential of social media to advance social justice initiatives and health equity, there remains a limited understanding of how effectively these platforms are utilized to promote critical consciousness among medical institutions.

Our study addresses this gap by exploring how institutional messaging by medical schools convey CC in their public outreach on X. Utilizing a mixed-method approach, including deductive content analysis and critical discourse analysis, we aim to characterize and evaluate the presence and impact of CC-related messaging. This effort seeks not only to quantify the extent of CC expression but also to qualitatively understand the messaging strategies employed. Through this analysis, we aim to offer insights into harnessing social media more effectively for promoting social accountability and justice within healthcare, aligning with the broader goals of medical education in fostering an equitable health system [16,17].

## Methods

We performed a deductive content analysis, followed by a critical discourse analysis of Canadian medical schools’ Twitter posts.

### Theoretical framework

Due to its focus on social justice and the growing push towards social justice in medicine, this study was guided by the principles of CC. Achieving CC can be broken down into three steps: awareness of the systemic inequalities and injustices, critical reflection on the causes of these inequalities, and actions at transforming the unjust system. Integrating CC into medical education is especially crucial to train healthcare professionals who are not only clinically competent, but also socially conscious and attuned to the disparities in healthcare. By analyzing messages of CC on social media, like Twitter, our study aims to better understand the how medical schools push for social justice in medicine.

### Data collection and analysis

First, all official Twitter accounts from medical schools in Canada were identified. Then, tweets posted over a 3-month period between March 22 and June 22, 2023 of each account were collected and downloaded using www.apify.com [18]. This time frame was selected by the authors as it offers a representative sample of the calendar year, encompassing the winter, spring, and summer seasons. Additionally, this period includes National Indigenous History Month, thereby capturing cultural observances relevant to the Canadian context.

### Content analysis

The deductive content analysis was applied in the first half of the study as a method to explore the different domains/types of critical consciousness (CC). In general, content analysis is a “systematic and objective means” of analyzing written, spoken, or visual communication. This is achieved by grouping similar linguistic strategies (such as specific words, phrases, etc.) into different groups to identify themes among the data. Deductive content analysis is specifically used when existing data is applied in a novel context. A codebook is first established based on previous data. This codebook outlines the rules upon which future data is coded and divided into separate categories [19].

In the deductive content analysis, the different subtypes of CC were identified by establishing a codebook. Based on previous literature, our codebook divided CC into 7 sub-categories: awareness of inequality, reflection of power dynamics, resistance to oppression, action towards change, employment of marginalized groups, understanding of systems of privilege, and intersectional analysis (See S1 Appendix). Each sub-category has specific words and/or themes. After creating the codebook, the tweets were chronologically coded as either displaying CC or not. If they showed CC, the tweets were further subcategorized into the different subdomains they displayed. Only the material that was visible on Twitter were analyzed (i.e., links to articles or videos were not reviewed). Furthermore, the data was coded by two independent coders with consistent comparison to minimize bias.

### Critical discourse analysis

Following the breakdown of CC into different subtypes, critical discourse analysis (CDA) was employed to understand how the language functions in a given text. Fairclough’s approach to CDA involves examining the social context under which a piece of text is produced, the means of distribution and consumption of this text, and finally the vocabulary and linguistic strategies that the text uses. By analyzing all three levels, meaning can be given to the text [20]. By using CDA in our study, we were able to more finely examine the communication strategies that were utilized within each subtype. Common language patterns and usages were uncovered and explored from the data.

### Ethical considerations

In accordance with Western University protocol and Tri-Council Policy Statement Article 2.2, this qualitative study was exempt from ethics board approval since no direct contact was made with users and it did not pose any risk to or adversely affect the welfare of any individuals. The website used in this study (Twitter, X) notified users of public access to all materials. Our team functioned solely as observers.

## Results

### Results from deductive content analysis

We identified 16 official medical school Twitter accounts across Canada, and from these 3442 tweets were collected and reviewed. The content analysis showed that 554 tweets displayed CC (16.12%).

**Fig 1:**
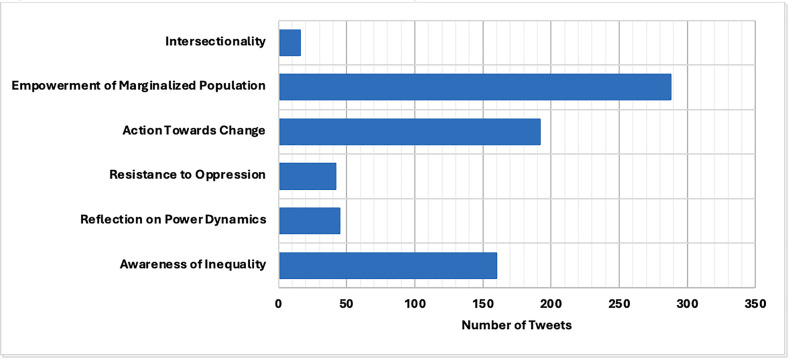
Number of Tweets within Each Sub-Category of Critical Consciousness. Empowerment of Marginalized Population had the highest number of tweets, followed by Action Towards Change, and Awareness of Inequality. There was a notable lack of tweets regarding Intersectionality.

The most prominent domain of CC was “Empowerment of Marginalized Populations” (n = 286 of all CC-related tweet). The second leading sub-type was “Action Towards Change” (n = 194), followed by “Awareness of Inequality” (n = 164). The next three categories were less prominent and included “Reflection on Power Dynamics” (n = 50),“Resistance to Oppression” (n = 43) and “Intersectionality” (n = 20) (See Fig 1)

**Fig 2:**
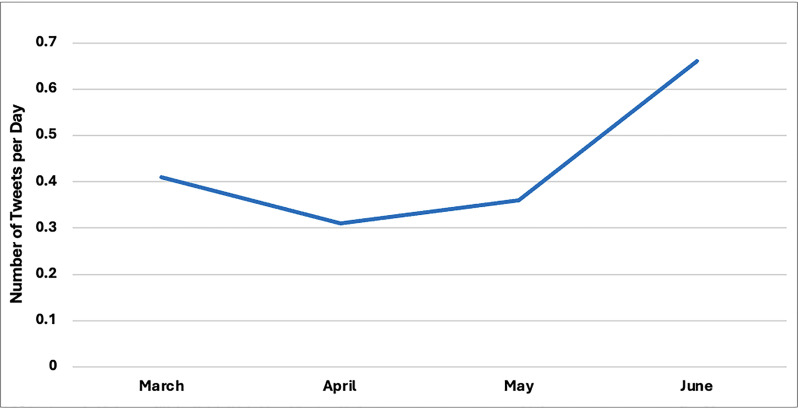
Number of Tweets that Displayed CC per Day in Each Month. June (n = 202) had the most tweets that displayed CC and March was the lowest (n = 60). May (n = 159) and April (n = 137) were second and third respectively. However, since the same number of days were not reviewed among every month, the data was corrected and displayed as the number of tweets that displayed CC per day.

We noted likely seasonal impact on the results with the highest number of tweets that demonstrated CC were posted in June, which is also Pride Month and National Indigenous History Month in Canada (n = 202) (See Fig 2)

### Critical discourse analysis

Within the guiding framework of critical consciousness, we found that schools predominantly leveraged Twitter to highlight marginalized groups, their accomplishments, and the challenges they face (critical awareness). These communities received positive spotlight by medical schools on X, drawing attention and awareness to the challenges they face and supporting them within Canada’s medical system. There was, however, a lack of critical reflection regarding institutions’ roles within systemic inequities. The discourse primarily externalized issues, positioning the schools and their followers as allies rather than focusing attention to institutions’ potential contributions to these disparities.

We have categorized the prominent language patterns in two overarching domains: “Instrument of Empowerment” and “Social Connection and Allyship”. We also reveal missing “Institutional Self-Reflection”

#### Language as an instrument of empowerment.

Perhaps most prominent within Canadian medical schools’ social media discourse, the use of language as an instrument of empowerment highlights initiatives that provoked change within medicine. Institutions commonly use X to praise their faculty who did novel work or research with marginalized populations, raising awareness of the struggles of these communities. The findings of these projects would be shared in a tweet with a link to access more information. This strategic deployment of language appeared to serve as a potent tool for fostering awareness and motivating action, key components of Critical Consciousness.

The language used to describe individual contributions frequently employs empowering verbs and adjectives that evoke a sense of action and positive change. Changemakers were positioned as” champions,” and “role models,” and many tweets emphasized the work of individuals from historically marginalized communities as “inspiring” and “positive change”.

*Congrats to new grad, _______ who co-founded the _______ Black Medical Student Association and has been a champion for diversity and equity on campus, seeks to create positive change in healthcare and for healthcare providers.*

*Congrats Dr. ____! You are a wonderful role model and great advocate for rural medicine!*

(in reference to this individual receiving an award for their contributions to rural medicine)

These terms and phrases of empowerment portrayed the subjects not only as participants but as proactive agents in the realm of social justice and health. These messages seemed to aim towards inspiring the academic community and broader public by showing tangible examples of how efforts lead to societal improvements, acknowledging the hard work and dedication of individuals and them individuals as role models for others within the institution. Beyond this purposeful vocabulary, hashtags like “#SocialAccountability” side-by-side with institutional hashtags further aligned the work of individuals as a key piece within the overall work of the school.

#### Language as a tool for social connection and allyship.

Canadian medical schools also leverage language to forge connections with diverse societal groups and position themselves as committed allies. We noted specific linguistic strategies during public holidays, recognition months, and cultural observances to not only celebrate diversity but also demonstrate an institution’s aim to promote inclusion. Common words such as “celebrates,” “recognizes,” and “honours” are consistently chosen to convey solidarity and support for marginalized groups, reinforcing the schools’ roles as inclusive environments.

*June is National Indigenous History Month and June 21 is National Indigenous Peoples Day, which recognizes and celebrates the history and diverse cultures of First Nations, Inuit, and Métis Peoples across Canada*

Moreover many tweets in this domain explicitly highlight an institution’s own action, for example during Pride Month. The use of vibrant and inclusive language is evident in tweets that extend beyond mere recognition to active celebration and support:

*“Happy #PrideMonth! [Institution] is dedicated to fostering a campus where everyone feels respected, valued, and included. During Pride Month, and the [local Pride event] in July, we’ll share resources and programming taking place at [Institution]. Stay tuned for more #Pride!”*

This tweet employs not just celebratory language but also phrases that communicate ongoing commitment and active participation (“dedicated to fostering,” “share resources and programming”), highlighting the institution’s role as an active ally in the LGBTQ+ community. By communicating in a manner that is inclusive and supportive, these institutions strengthen their societal connections and social capital, reinforcing their commitment to allyship.

The language used in observance of cultural recognition months also plays a significant role in how institutions connect with and educate their communities about specific cultures. Many such tweets encouraged additional educational engagement (“Learn more”), promoting a deeper understanding and appreciation within and beyond the institution. A tweet about Jewish Heritage Month exemplifies this approach: *“May is Jewish Heritage Month. It recognizes the culture and significant contributions of the Jewish community to Canadian society. Learn more about the Jewish community in Canada”*

This was also observed in another tweet in remembrance of a local tragedy, “*Today we remember [local tragedy] and stand in solidarity with our Muslim Community against Islamophobia. Thank you to [local library] for curating reading lists highlighting Muslim authors, experiences, and themes of empathy and compassion.”*

Furthermore, contrasting the positive messages of praise and celebration, schools also used negative imagery of battle and violence to display critical consciousness and show support and allyship by reminding the public of the historical atrocities committed against marginalized populations.

*Join us on May 5th to remember and honor the missing and murdered Indigenous women and girls. Let’s stand in solidarity with our Indigenous communities and wear red to raise awareness. Video by tanookialex, Indigenous artist.* #*MMIWG* #May5th #NationalDayofAwareness

With language that evoked feelings of violence, schools personified marginalization and created a battle between themselves and inequity. Coupled with messages of “solidarity” and “rememb[rance]”, these tweets paint a united front between the schools, the audience, and the marginalized groups. This dichotomy of messages was effective at communicating the importance of celebrating underrepresented communities while also not forgetting their histories.

#### Institutional self-reflection.

While Canadian medical schools adeptly use Twitter to spotlight individual achievements and align with diverse communities, there is a lack of tweets critically reflecting on the institutions’ own roles within systemic or structural inequities. Across all tweets the discourse around social justice was often limited in the extent to which medical schools acknowledge their positions of power.

Our analysis shows that tweets celebrating achievements and societal connections rarely engage in a deep introspection of the schools’ own systemic contributions to the issues discussed. The language used often portrays the institutions as bystanders or supporter of change, rather than as active participants needing to evolve internally. For instance, tweets frequently commend external initiatives and individual contributions to social justice and inclusivity but fall short of acknowledging or mentioning the schools’ own policies or historical legacies that contribute to ongoing disparities.

Instances where institutions hint at a need for internal change often lack depth or specificity. Vague mentions of “ongoing efforts” or “commitment to improvement” are common but rarely detail what these efforts entail or how the institutions plan to address their past failures. For example:

*During National #IndigenousHistoryMonth, join us in commemorating the rich cultures and histories of the First Nations, Inuit and Métis Peoples. #NIHM We invite everyone in our community to participate in special activities organised by [Institution]*

While this statement suggests a reflective stance, it does not engage critically (within the body of the tweet nor via external link) with the specifics of institutional reform or the structural changes necessary for genuine transformation. Such statements are not substantial, as they do not provide transparency or accountability.

*Dr. _____ will introduce learners to the concept of ableism, that is disability prejudice & discrimination, as endemic to society as a whole, & to healthcare.*

**Fig 3:**
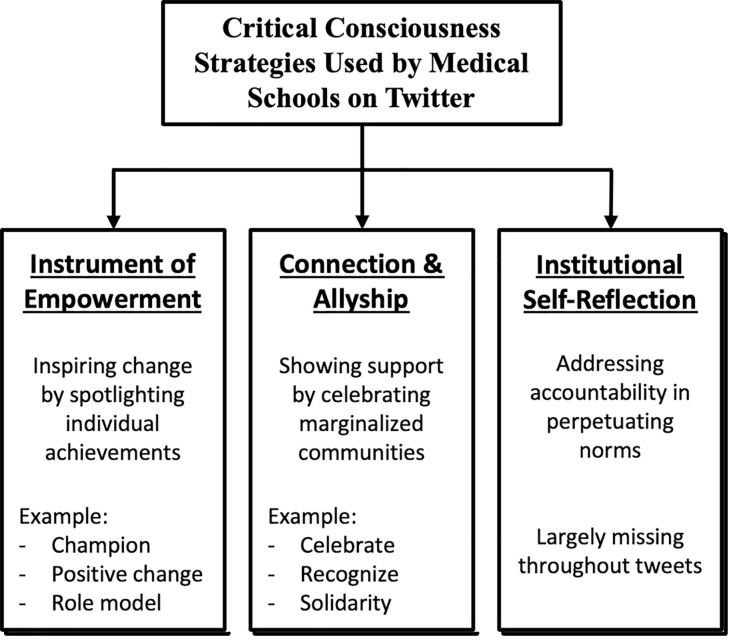
Number of tweets that displayed CC per day in each month.

While potentially constructive, this tweet pushes the responsibility onto individuals and lacks a framework for how the institution will implement changes based on this feedback or measure its impact. Across all tweets language tends to not address institutional accountability, emphasizing external actions over internal changes, suggesting a lack of critical reflection. A visual summary of the critical consciousness techniques used by medical schools can be found in Fig 3.

## Discussion

In our analysis of Canadian medical schools’ X communications through the lens of Critical Consciousness (CC), we observed a pronounced focus on raising awareness about individual accomplishments from members of historically marginalized group and maintaining a social alignment with these communities. This emphasis on awareness, while crucial, was not matched by reflections or demonstration of accountability on the institutions’ roles in these systemic inequities or actionable steps towards addressing them. The significance of these findings is that social media is being underutilized by institutions; as noted by Murthy, platforms like X can be used to showcase a school’s commitment to the transformative aspects of critical consciousness and serve as a space for activism [15]. By struggling to move beyond awareness, there is a lack of deeper engagement with social justice and transformative action. This form of discourse that display is not confined to X; Brown et al. analyzed 45 public statements from academic medical organizations in Canada and USA following the murder of George Floyd in 2020 and found similar themes of a lack of critical self-reflection and institutional accountability when it comes to addressing anti-Black racism in academic medicine. In this study, the discourse employed by medical schools portrayed anti-Black racism as “outside the institution”, distancing the organizations from responsibility in maintaining or reinforcing problematic norms and effectively downplaying anti-Black racism in medicine. Instead of highlighting the necessary structural changes needed in academic medicine, institutions call for actions at an individual level through interventions like “bias training”, effectively distancing themselves from racism as a broad social issue [2]. Such consistent findings reiterate the need for institutions to towards transformative action.

The notable absence of institutional self-reflection in online messages has not gone unnoticed by advocacy groups representing marginalized voices. For example, the Black Medical Students’ Association of Canada tweeted:

*Performative #allyship is a thing.*

*Being anti-racist is not a couple of retweets and shares on your social media.*

*TIME, LEARNING, ACTION in your communities, STANDING UP beside us in protest, SPEAKING AGAINST those around you who are racist.*

This public critique displays the significance of these findings: marginalized populations are dissatisfied with current messaging and demand tangible engagement online. Such posts reflect broader frustration with what is perceived as institutional reluctance and a controlled form of communication, rather than genuine responsiveness.

The predominance of awareness-focused content over reflection and action points to a critical gap in the use of social media for social accountability efforts. Previous literature emphasizes the role of higher education institutions in not just acknowledging social injustices but actively participating in the dismantling of these structures through self-examination and systemic change [1]. The framework of CC, rooted in the work of Paulo Freire, underscores the importance of moving beyond awareness to critical reflection and action [21].

Our observations suggest a missed opportunity. Despite reflective conversations potentially being held internally, a lack of critical self-reflection in public messaging may hinder the effectiveness of social justice initiatives and impact the trust marginalized communities have in medical schools as agents of societal change [22]. Social media platforms like Twitter offer a unique space for institutions to communicate their ongoing social justice work, and such discussions could serve as a powerful tool for transparency and accountability, demonstrating a commitment to not only identifying problems but also actively working on solutions [23]. As argued by Ahmed [22], the real challenge for institutions lies in moving beyond the surface-level engagement with diversity, equity, and inclusivity (DEI) issues to embedding these principles into the fabric of their organizations.

To leverage social media effectively, medical schools could adopt a more holistic approach to communicating their social accountability efforts. Institutions could use their platforms to report not only on their successes but also on their challenges and failures in addressing social justice. This transparency can help build trust and demonstrate a genuine commitment to change [24]. Social media should be used as a tool to engage in meaningful dialogue with both the internal community and the broader public. This includes discussing uncomfortable truths about the institutions’ past and present roles in the inequities present and soliciting feedback on how to improve [23]. By doing so, these institutions can use their platforms to model a comprehensive engagement with DEI principles, encouraging a culture of accountability, free of tokenism, and continuous improvement within the medical education community and beyond [3].

Comparing our findings with studies from other sectors, such as corporate social responsibility initiatives, suggests that challenges in authentically engaging with social justice are not unique to medical education. Research indicates that across various fields, institutions struggle with moving beyond superficial gestures to implement substantial changes [3]. However, unlike corporate settings, medical schools operate within a context where the impact of their actions—or inactions—directly affects health outcomes, making their commitment to CC particularly crucial [4]. The COVID-19 pandemic, a time where medical schools harnessed social media platforms for rapid data dissemination and online dialogues, showed a 72% increase in tweet impressions and a near doubling of profile visits for medical journals. This scale of engagement by institutional messages proves the influence that medical schools have in their digital outreach and how effective dissemination of public health messages can change behaviour [25].

Our results should not be taken as a criticism of institutions’ social media and communications teams; rather, these findings should provoke reflective discussions around how institutions in power (i.e., medical schools) could use social media more intentionally. Social media use in medicine is primarily driven by a risk-averse mentality; there is great anxiety around one’s online presence and how that will jeopardize their careers and patient privacy. This is especially true with medical schools, an institution that represents a host of interests from students to attending physicians. While DEI is of utmost importance in online discussions, medical school must also balance the interests of their marketing and legal teams when posting content. Regardless, healthcare must move beyond such hesitancies and use platforms such as Twitter intentionally to actually improve the health of our patients [26].

The reluctance of Canadian medical schools to meaningfully engage with DEI principles may reflect the internal cautious policies when posting social media messages which are not publicly available. However, these findings suggest the need for Canadian medical schools to either develop transparent guidelines that communicate their DEI initiatives or update current policies. This includes defining how institutions engage in critical self-reflection online, address public concerns raised via social media, and how they report on DEI progress. Social media messaging is a relatively new phenomenon in the agenda of medical schools; however, embedding such practices will enhance accountability and elevate public trust in medical institutions as true agents of change towards health equity.

Our study was limited by the single time-window of data collection (3 months in 2023) which may not be reflective of annual use. Moreover our data focused on Canadian medical schools, and the sociopolitical context may differ from healthcare institutions in other countries. We analyzed user-facing content on Twitter, i.e., tweets, and not the entirety of content in external links. This represents a challenge as tweets are primarily perceived as a short form of messaging which may be an obstacle when attempting to engage in complex self-reflection discussions. Given the widespread impact of social media and the role of academic institutions in shaping discourse around medicine and healthcare [6], these results may still have broad hypothesis-generating implications for wider audience. Additionally, our study did not analyze public responses, critiques, or comments to institutional tweets, nor did we examine tweets from individual members of medical schools. These perspectives represent important areas for future research to explore how institutional messaging is perceived, challenged, or supplemented by both the public and institutional affiliates. Future research can collate and analyze responses to tweets by Canadian medical schools to understand potential resonance or resistance to institutional messaging regarding critical consciousness and health equity. Future work can also assess the social media posts of members of medical schools, who often have greater freedom to participate in critical dialogue online, to identify discrepancies between institutional messaging and the voices of staff and understanding how individuals engage with social accountability, highlighting the broader culture within the institution.

In conclusion, while Canadian medical schools are making important strides in raising awareness about social justice issues on Twitter, however, there is ample room for these communications to more fully embrace the principles of critical consciousness by engaging more deeply and authentically in critical reflection. By doing so, they can more effectively contribute to the creation of a more equitable and just medical education system and healthcare landscape.

## Supporting information

S1 File Tweets extracted from each medical school and their corresponding code. (XLSX)

S1 Appendix Codebook used for categorizing tweets into different CC domains. (DOCX)
