# Supplementary material for: Social justice and social media: How medical schools display critical consciousness online
Source: PLOS Digit Health. 2025 Aug 7;4(8):e0000981. doi: 10.1371/journal.pdig.0000981 (PMC12331073; doi:10.1371/journal.pdig.0000981)
Supplement: S1 Appendix — (DOCX) [file pdig.0000981.s002.docx]

**S1 Appendix: Summary of Codebook for Critical Consciousness**

| **Domains of Critical Consciousness** | **Definition in Codebook** |
| --- | --- |
| Awareness of Inequality | This sub-type represents tweets whose sole aim is to raise awareness of inequality and no other code. This can be achieved by referencing the structural inequalities among different social, economic, and/or racial groups. |
| Reflection on Power Dynamics | This sub-type of CC sought to describe tweets that were socially critical and that exposed the power dynamics that related to systems of privilege and oppression. |
| Resistance to Oppression | The Resistance to Oppression sub-type of CC was coded for schools that demonstrated resistance, which could have been accomplished by acts of solidarity or protests against unjust policies. Tweets coded for this sub-type shared a common theme of raising awareness about violent behaviours against a marginalized group. |
| Action Towards Change | The Action Towards Change sub-domain of CC describes tweets that promote readers to change oppressive systems and structures |
| Empowerment of Marginalized Populations | This sub-type of CC involved tweets that uplifted marginalized communities, which can be achieved through various ways. |
| Intersectionality | The criteria for intersectionality were tweets that referred to the compounding effect of multiple forms of oppression and privilege. Tweets coded in this section brought forth the importance of intersectional analyses. |
